# Clustering Cloud-Like Model-Based Targets Underwater Tracking for AUVs

**DOI:** 10.3390/s19020370

**Published:** 2019-01-17

**Authors:** Mingwei Sheng, Songqi Tang, Hongde Qin, Lei Wan

**Affiliations:** Science and Technology on Underwater Vehicle Laboratory, Harbin Engineering University, Nantong Street 145, Harbin 150001, China; smwsky@hrbeu.edu.cn (M.S.); jeremytangsq@163.com (S.T.); wanlei@hrbeu.edu.cn (L.W.)

**Keywords:** AUV, data association, clustering cloud-like model, underwater objects tracking

## Abstract

Autonomous underwater vehicles (AUVs) rely on a mechanically scanned imaging sonar that is fixedly mounted on AUVs for underwater target barrier-avoiding and tracking. When underwater targets cross or approach each other, AUVs sometimes fail to track, or follow the wrong target because of the incorrect association of the multi-target. Therefore, a tracking method adopting the cloud-like model data association algorithm is presented in order to track underwater multiple targets. The clustering cloud-like model (CCM) not only combines the fuzziness and randomness of the qualitative concept, but also achieves the conversion of the quantitative values. Additionally, the nearest neighbor algorithm is also involved in finding the cluster center paired to each target trajectory, and the hardware architecture of AUVs is proposed. A sea trial adopting a mechanically scanned imaging sonar fixedly mounted on an AUV is carried out in order to verify the effectiveness of the proposed algorithm. Experiment results demonstrate that compared with the joint probabilistic data association (JPDA) and near neighbor data association (NNDA) algorithms, the new algorithm has the characteristic of more accurate clustering.

## 1. Introduction

Most research concerning automatic detection and tracking technology is focused on ground objects. However, underwater multi-target detection and tracking are in great demand. The past decade has witnessed the fast development of ocean observation, which has been a success in the application of subaqueous robots for underwater detection and salvage, target recognition, and location tracking [1,2,3,4]. Underwater multi-target detection relies on underwater multiple sensors [5]. Based on the mechanically scanned imaging sonar, the multi-target detection and tracking technique can extract information from one or several specific moving objects in the intricate background area through a series of sonar sequence images, which can automatically track the targets [6,7].

The special underwater operation environment has been a hindrance to the development of marine resources, requiring professional underwater tools. On account of the limitation of human underwater activities, it is necessary to develop an unmanned vehicle that can replace divers and work under deeper water in order to develop marine resources. Various underwater robots have emerged as the times have required [8]. As a sort of subaqueous operation device, autonomous underwater vehicles (AUVs) can complete underwater operations under the condition of unmanned operation, and can fulfill the tasks of environmental detection, target recognition, and so on [9,10,11]. With the continuous development of related technologies and theories in recent decades, AUV technology is constantly upgrading, and its application to complete underwater detection is low-cost and highly-efficient. As a result, the utilization of AUVs is more extensive in tasks such as marine resources exploration and marine environment survey [12,13,14].

When the relative position of the object’s acoustic reflection surface varies, the intensity of the emission echo received by the sonar weakens and the target may disappear. Because the mechanically scanned imaging sonar is two-dimensional, the sonar image of the target area will overlap [15,16,17] when the two targets are in the same sphere as the sonar beam, making it hard to track the target based on the mechanically scanned imaging sonar. Especially in the dense multi-target tracking, the target density and the overlapping of the target regions in the sonar image will make the target tracking rather difficult. The basic principle of data association is to settle the problem of mutual matching between the trace points based on logic judgment or probability statistics calculation.

The common data association algorithms include the nearest neighbor data association [18], the probability data association [19], the joint probability data association [20], the multiple hypothesis tracking algorithm [21], and the clustering method [22]. L. Zhang [23] proposed an optimization method on the basis of network flow for data association in a multi-target tracking process. The maximum a posteriori (MAP) problem is mapped to the cost flow network with trajectories and non-overlapping constraints. Moreover, the optimal data association is figured out using the minimum cost flow algorithm in the network. J. Neira and J.D. Tardos [24] from Zaragoza University raised a new set of measurement systems with joint compatibility, which refuse to be misaligned, and the measurement systems employ a restrictive standard to search for the best solution for data association efficiently.

Although some approaches have been currently presented, problems still exist. The object-tracking method based on the extended Kalman filter (EKF) [25,26] has a higher computational efficiency, and the perfect track results can be gained under the condition of linearity motion. An alternative algorithm applying the Sequential Monte Carlo method has the advantage over solving the nonlinearity problems in the measurement model. However, the large numbers of particles lead to a higher computational cost than EKF [27,28].

The rest of the study is organized as follows: Section 2 presents the hardware architecture of the multiple targets tracking system of AUVs. The CCM algorithm is applied to cluster the effective echoes to the targets, and the cluster center is taken as the final observed value. Furthermore, the nearest neighbor method is involved in associating the cluster center with the track and state estimation to form a new multi-target data association algorithm. The proposed multi-target data association algorithm based on the C-means clustering cloud-like model is outlined in Section 3. The sea trial of the mechanically scanned imaging sonar equipped on “Orange Shark” AUV is implemented so as to verify the effectiveness of the proposed algorithm, and the experimental results and a brief discussion are shown in Section 4. Finally, conclusions and future works are presented in Section 5.

## 2. Hardware Architecture

**Figure 1:**
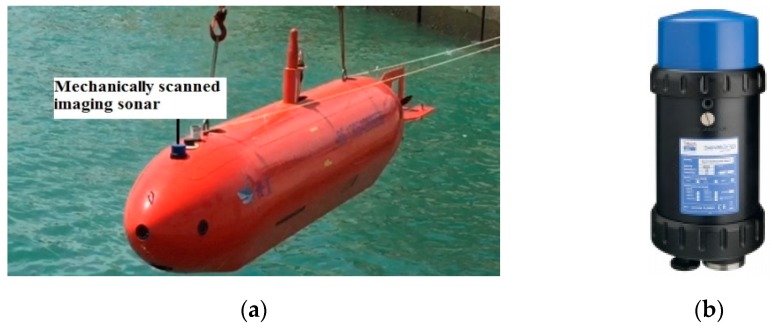
“Orange Shark” autonomous underwater vehicle (AUV) with its mechanically scanned imaging sonar. (**a**) “Orange Shark” AUV, (**b**) Mechanically scanned imaging sonar.

In the targets underwater tracking system of AUVs, mechanically scanned imaging sonar is mounted on an “Orange Shark” AUV (Figure 1a) in order to track the multi-target in real marine environment. The parameters of the Super SeaKing DST forward-looking sonar (Figure 1b [29]) are shown in Table 1, which hosts two mechanically scanned imaging sonars in a single subsea pressure housing; a 325 kHz CHIRP sonar with a true operational range of up to 300 m for long range target recognition, and a 650 kHz CHIRP sonar for ultra-high definition images. Low frequency mode is chosen for a faster scan speed of multiple targets tracking.

**Figure 2:**
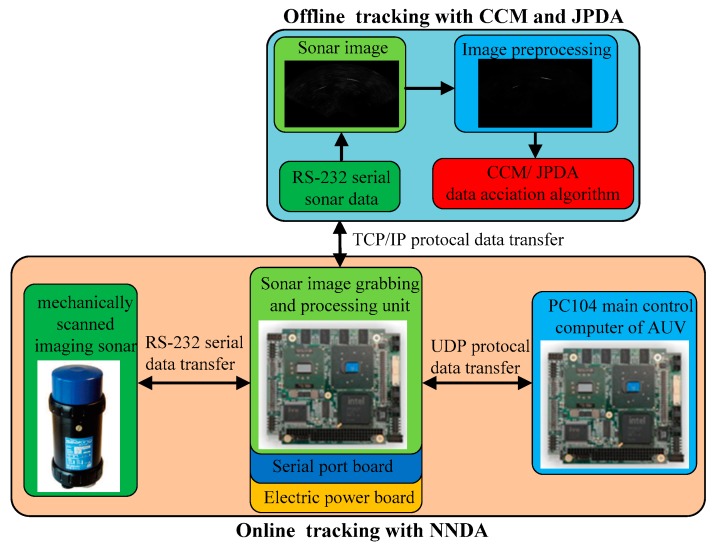
Block diagram of underwater targets tracking for AUVs.

The block diagram of underwater targets tracking for AUV is presented in Figure 2. The sonar image grabbing and processing unit mainly consists of three parts, a PC104 sonar image processing computer, a PC104 main control computer of the AUV, and the mechanically scanned imaging sonar. The PC104 sonar image processing computer transfers serial data via the RS-232 serial port. The serial data is saved in local hard disks and is transmitted to sonar images in the PC104 sonar image processing computer. When the multi-target positions are predicted by adopting the data association algorithm, the positions are transferred to the PC104 main control computer of the AUV via the User Datagram Protocol (UDP). The AUV launches to track multi-targets according to the trajectory planning as soon as receiving the multi-target positions. The near neighbor data association (NNDA) algorithm is used to conduct an online multi-target tracking test in the AUV, and the test data is recorded in PC104. The mechanically scanned imaging sonar data is exported from the AUV and the target tracking data processing is performed by the joint probabilistic data association (JPDA) and clustering cloud-like model (CCM) algorithm offline. The test results are compared so as to verify the underwater target tracking accuracy.

**Figure 3:**
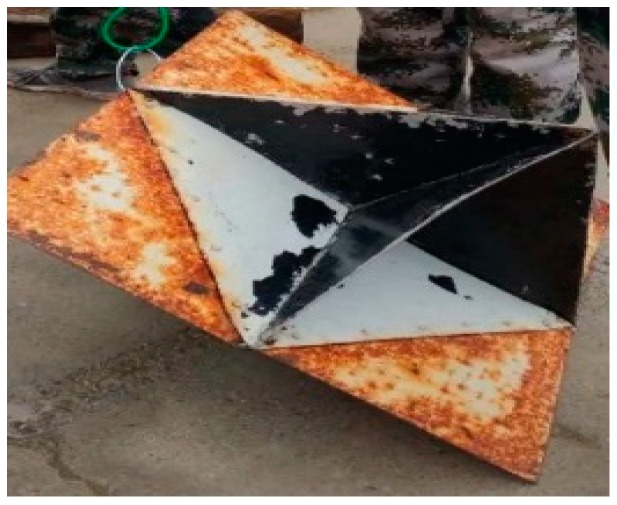
Target 1: a corner reflector (50 mm × 50 mm × 50 mm).

**Figure 4:**
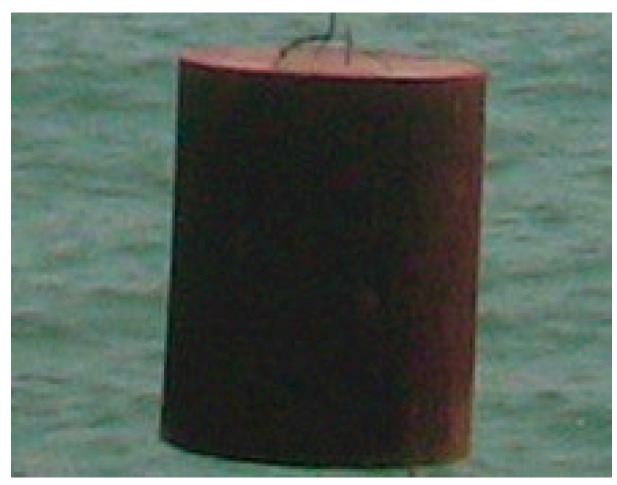
Target 2: a cylinder reflector (diameter: 50 mm; height: 80 mm).

During the sea trail experiments, two targets were planted in the sea, and the selected targets with different underwater acoustics reflectivity are shown in Figure 3 and Figure 4.

## 3. Methodology

Data association is a significant process for underwater multiple targets tracking. To settle the issue of data association in the multi-target tracking, a data association algorithm in line with the clustering cloud-like model is proposed.

### 3.1. The Clustering Algorithm Based on the Cloud-Like Model

The cloud-like model combines the fuzziness and randomness of the qualitative concept, which can achieve the conversion of the qualitative linguistic values and quantitative values. Moreover, the model can change the qualitative language value into the quantitative value, which is the cloud-like droplet of the model. During the transformation, the cloud-like droplet generation is a random event, and thus it can be described by the probability distribution.

The traditional C-means clustering issue can be described as follows: *P*-dimension samples {*x*_1_, *x*_2_, …, *x*_n_} can be divide into *c* classes, and each class is represented by a clustering center *v_i_* (*i* = 1, 2, …, *c*). The degree of membership of the sample in each category can be represented by a classification matrix *U* = [*u_ik_*]*_cn_* (where *u_ik_* represents the degree of membership of the *k*-th sample for class *I* and $0\leq u_{ik}\leq 1$). The clustering aims to find the optimal *U* and $V=[v_{i}]$ for minimizing the objectives function J(U,V). In the fuzzy C-means clustering method (FCM), we computed the membership function as follows:

(1) $$\mu_{i}(x_{k})=u_{ik}=\frac{{\left|\left|x_{k}-v_{i}\right|\right|}^{\frac{2}{m-1}}}{\sum_{j=1}^{c}{\left|\left|x_{k}-v_{j}\right|\right|}^{\frac{2}{m-1}}}$$

where || . || is the suitable norm and *m* is the fuzzy weighted index.

In the C-means clustering method based on the model, each class is represented by a cloud-like model. The cloud-like expectation is regarded as its clustering center. The classification matrix is calculated by the mean of the certainty degree of the samples in the cloud-like model. During the transformation, the cloud-like droplet generation is a random event. Therefore, it can be described by the probability distribution. The cloud-like model principally contains the following three digital features: expected value (*E_x_*), entropy (*E_n_*), and hyper entropy (*H_e_*). The expected value (*E_x_*) refers to the expected value of a cloud-like droplet on the spatial distribution of the domain, which remains the most representative value of the qualitative concept. Generally, the greater the entropy (*E_n_*) is, the more macroscopic the concept will be in the cloud-like model. Additionally, it is determined by the randomness and fuzziness of the concept. *E_n_* is the measurement of the qualitative concepts of randomness, reflecting the dispersion degree of the cloud-like droplets that represent this qualitative concept. Furthermore, it is the measure of the qualitative concepts of fuzziness, which signifies the value range of the cloud-like droplets accepted in the domain. The measurement of the hyper entropy’s (*H_e_*) uncertainty, which is the entropy of the entropy, is jointly decided by the randomness and fuzziness of the entropy, reflecting the condensation degree of the cloud-like droplets.

Supposing (*E_x_*, *E_n_*, and *H_e_*) is the *i*-th class *P*-dimension cloud-like model, and *E_x_*, *E_n_*, and *H_e_* is a *P*-dimension vector, respectively. The clustering center is $v_{i}=E_{ei}$ and the elements of the classification matrix are Equation (2).

(2) $$u_{il}=\overset{p}{\underset{i=1}{\wedge }}\exp[-\frac{{\left(x_{il}-Ex_{il}\right)}^{2}}{En_{il}^{2}}]$$

where $x_{il}$, $E_{x_{il}}$, and $E_{n_{il}}$ are the *l*-dimension elements of $x_{i}$, $E_{x_{i}}$, and $E_{n_{i}}$, respectively.

The clustering objective function of CCM is similar to FCM, and the clustering objective function is as follows:

(3) $$J(U,V)=\sum_{i=1}^{c}\sum_{k=1}^{n}u_{ik}^{m}{\|x_{k}-v_{i}\|}^{2}$$

Under a constraint condition (the normalization condition $\sum_{i=1}^{c}u_{jk}=1$), FCM gets the iterative formula adopting the iterative solution through the Lagrange multiplier method. Without the above constraints, the objective function of CCM is computed using the iteration of the forward and reverse cloud-like transformation. Considering that the sample set for clustering here is the underwater target sonar image data, there is the random uncertainty (each sample is subject to some unknown probability distribution) and fuzzy uncertainty (each sample is a small sample set). The cloud-like model can depict fuzziness and randomness simultaneously, and thus the C-means clustering on the basis of the cloud-like model is involved in the data association so as to avoid the dependence of the clustering algorithm on the normalization conditions.

### 3.2. Multi-Target Data Association Algorithm Based on the C-Means Clustering Cloud-Like Model

Based on the above section, this section will cluster all of the valid echoes (targets in the sonar image) by the clustering algorithm using the cloud-like model. Then, the nearest neighbor method is involved to associate the targets. The cluster centers of the multiple targets are regarded as the final measurement for the given target for the state estimation. Supposing there are *m* echoes and *t* targets, the procedure is as follows:

(1) At the beginning of the track update cycle, the basic information concerning the prediction state vector ${\overset{\wedge }{X}}_{j}(k+1|k)$, the prediction measurement vector ${\overset{\wedge }{Z}}_{j}(k+1)$, the innovation covariance $S_{j}(k+1)$, and the gain $K_{j}$ of the target j (j=1,2,…,t) can be computed by Equations (4) to (8).

(4) $$\overset{\wedge }{X_{j}}(k+1|k)=F_{j}\overset{\wedge }{X_{j}}(k|k)$$

(5) $$\overset{\wedge }{Z_{j}}(k+1|k)=H_{j}\overset{\wedge }{X_{j}}(k+1|k)$$

(6) $$P_{j}(k+1|k)=F_{j}P_{j}(k|k)F_{j}^{\prime }+Q_{j}(k)$$

(7) $$S_{j}(k+1)=H_{j}P_{j}(k+1|k)H_{j}^{\prime }+R_{j}(k+1)$$

(8) $$K_{j}=P_{j}(k+1)H_{j}^{\prime }S_{j}^{-1}(k+1)$$

where $F_{j}$ is the state transition matrix, $H_{j}$ is the measurement matrix, $\overset{\wedge }{P_{j}}(k+1|k)$ is the prediction covariance, $Q_{j}(k)$ is the process noise covariance matrix, and $R_{j}(k)$ is the measurement noise covariance matrix.

(2) Before performing the multiple targets data association calculation, we should check if all of the measurements fall into the tracking wave gate. If ${v^{\prime }}_{ij}\left(k+1\right)S_{j}^{-1}(k+1)v_{ij}(k+1)<\gamma$, it is regarded as a valid target, where γ is the threshold value of tracking the wave gate. $v_{ij}(k+1)$ is the measuring information about measurement *i* to target *j*, as given in Equation (9), as follows:

(9) $$v_{ij}(k+1)=Z_{j}(k+1)-\overset{\wedge }{Z_{j}}(k+1|k)$$

where $\overset{\wedge }{Z_{j}}(k+1|k)\left(i=1,2,\ldots ,m_{k}\right)$ is the measurement acquired from the sonar.

(3) The CCM algorithm is presented to cluster the effective targets, which fall into the tracking wave gate in order to obtain the center of the cluster $V_{i}(k+1)$. The calculation process is as follows:

① We initialize *U*(0) and $U(0)={\left|u_{ij}^{0}\right|}_{t\times m_{k}}$, where

(10) $$u_{ij}^{0}=e^{-{v^{\prime }}_{ij}(k+1)S_{j}^{-1}(k+1)v_{ij}(k+1)}$$

② According to the rule of maximum degree of membership, *U*^(*b*)^ is utilized to classify the target effective targets {*Z*_1_(*k* + 1), *Z*_2_(*k* + 1),…, *Z_mq_*(*k* + 1)} into *t* classes. For each class of measurement targets, the cloud-like model $\left(E_{x_{jl}}^{\left(b\right)},E_{n_{jl}}^{\left(b\right)},H_{e_{jl}}^{\left(b\right)}\right)$ in the *b*-th dimension is calculated by a non-deterministic inverse cloud-like generator, where *j* = 1, 2, …, *t* and *l* = 1, 2, …, *p*. 

③ A new classification matrix $U^{\left(b+1\right)}=\left|u_{ij}^{{\left(b+1\right)}_{i}}\right|$ is calculated in accordance with each cluster of the cloud-like model, as shown in Equation (11), as follows:

(11) $$u_{ij}^{b+1}=\overset{p}{\underset{l=1}{\Lambda }}\left\{\exp\left[-\frac{{\left(Z_{il}(k+1)-E_{x_{jl}}^{\left(b\right)}\right)}^{2}}{2{\left(E_{n_{jl}}^{\left(b\right)}\right)}^{2}}\right]\right\}$$

where *Z_il_*(*k* + 1)*(l* = 1, 2, …, *p*) is the *l*-th dimension of the *i*-th measurement information; $E_{x_{jl}}^{\left(b\right)}$ is the *l*-th dimension of the mean of the *j*-th cloud-like model, and it is a random number generated from the *l*-th dimension’s the entropy $E_{n_{jl}}^{\left(b\right)}$ and the super-entropy $H_{e_{jl}}^{\left(b\right)}$ of the *j*-th cloud-like model.

④ We have the clustering objective function as follows:

(12) $$J(U^{\left(b+1\right)},V^{\left(b+1\right)})=\sum_{j=1}^{t}\sum_{i=1}^{mk}(u_{ji}^{\left(b+1\right)}{)}^{m}\|Z_{i}(k+1)-E_{x_{j}}^{\left(b\right)}\|$$

where $V^{\left(b+1\right)}=|E_{x_{j}}^{\left(b\right)}|$ is a matrix composed of each of the cloud-like model’s mean vectors.

⑤ If $\left|J(U^{\left(b+1\right)},V^{\left(b+1\right)})-J(U^{\left(b\right)},V^{\left(b\right)})\right|<\varepsilon$ (ε is a predetermined threshold), then the clustering is achieved and $V_{j}^{\left(k+1\right)}=E_{x_{j}}^{\left(b\right)}$. Otherwise, suppose *b* = *b* + 1 and return to step ①.

(4) The nearest neighbor algorithm is involved to find the cluster center paired to each target trajectory, and the cluster center is regarded as the final measurement of the corresponding target at time *k* + 1 (final stage of the track up-date cycle).

(5) The state and covariance of each target can be filtered and updated as follows:

(13) $$N_{j}(k+1)=V_{j}(k+1)-\overset{\wedge }{Z}(k+1|k)$$

(14) $$\overset{\wedge }{X_{j}}(k+1|k+1)=\overset{\wedge }{X_{j}}(k+1|k)+K_{j}N_{j}$$

(15) $$\overset{\wedge }{P_{j}}(k+1|k+1)=\overset{\wedge }{P_{j}}(k+1|k)+K_{j}H_{j}P_{j}(k+1|k)$$

## 4. Sea Trail Experiment Results and Discussion

**Figure 5:**
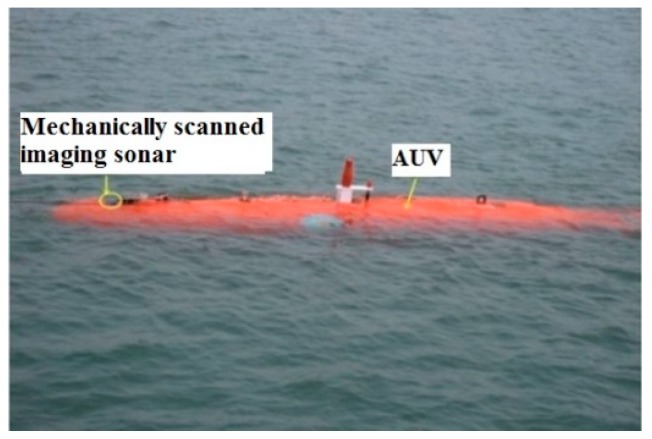
“Orange Shark” AUV.

**Figure 6:**
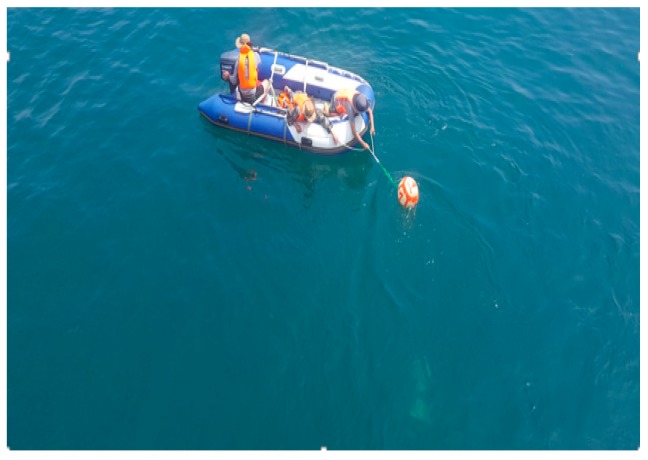
A target is dragging by a boat.

**Figure 7:**
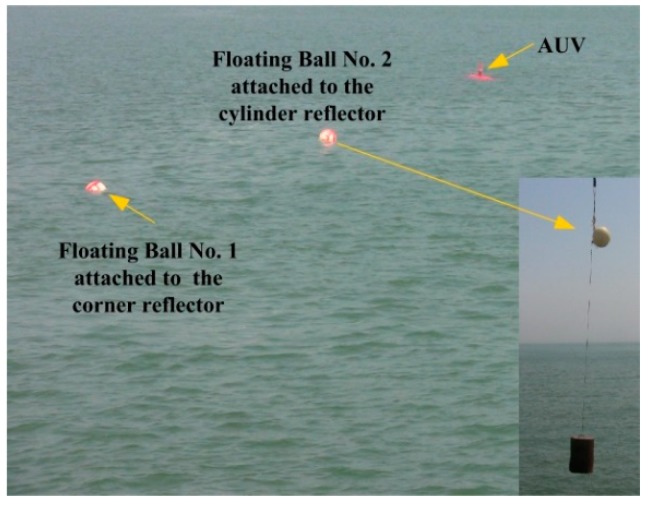
AUV tracking experiment scenario.

In order to verify the feasibility of the proposed algorithm based on the clustering cloud-like model in the actual underwater mission, a series of sea trail experiments have been implemented. The experiments were conducted in coast of the Yellow Sea. Two reflectors were located 3 m underwater, and the distance of the targets to the seabed was nearly 10 m. The mechanically scanned imaging sonar was fixedly mounted on an “Orange Shark” AUV. The sonar scanning distance of the mechanically scanned imaging sonar was set to be 50 m. In order to shorten the scanning time of the sonar, the scanning angle was set to 120 degrees forward. Several ropes attached to the associated targets were heaved to drive the targets moving in the horizontal and vertical directions. The AUV and experiment scenario are shown in Figure 5, Figure 6 and Figure 7.

The online tracking experiments using the NNDA of this paper were developed in Tornado 2.2 using C++, and were conducted on a PC104 with an Intel Pentium M 2 GHz CPU and 512 MB RAM; the operating system was Vxworks5.0. The computational power of the embedded PC104 bus system was a real limitation for using more complex algorithms, so offline tracking experiments adopting CCM and JPDA were developed in visual studio 2010 using C++, and were conducted on a PC with Intel Xeon 3.4 GHz and 8.00 GB RAM.

This section selects two sets of representative AUV sea trail experiment data to verify the tracking effect of the CCM algorithm, and compares it with the conventional nearest neighbor data association algorithm (NNDA) and the joint probabilistic data association (JPDA).

### 4.1. Results and Analysis of Non-Cross-Moving Experiment of Multiple Targets Experiment Using AUV

**Figure 8:**
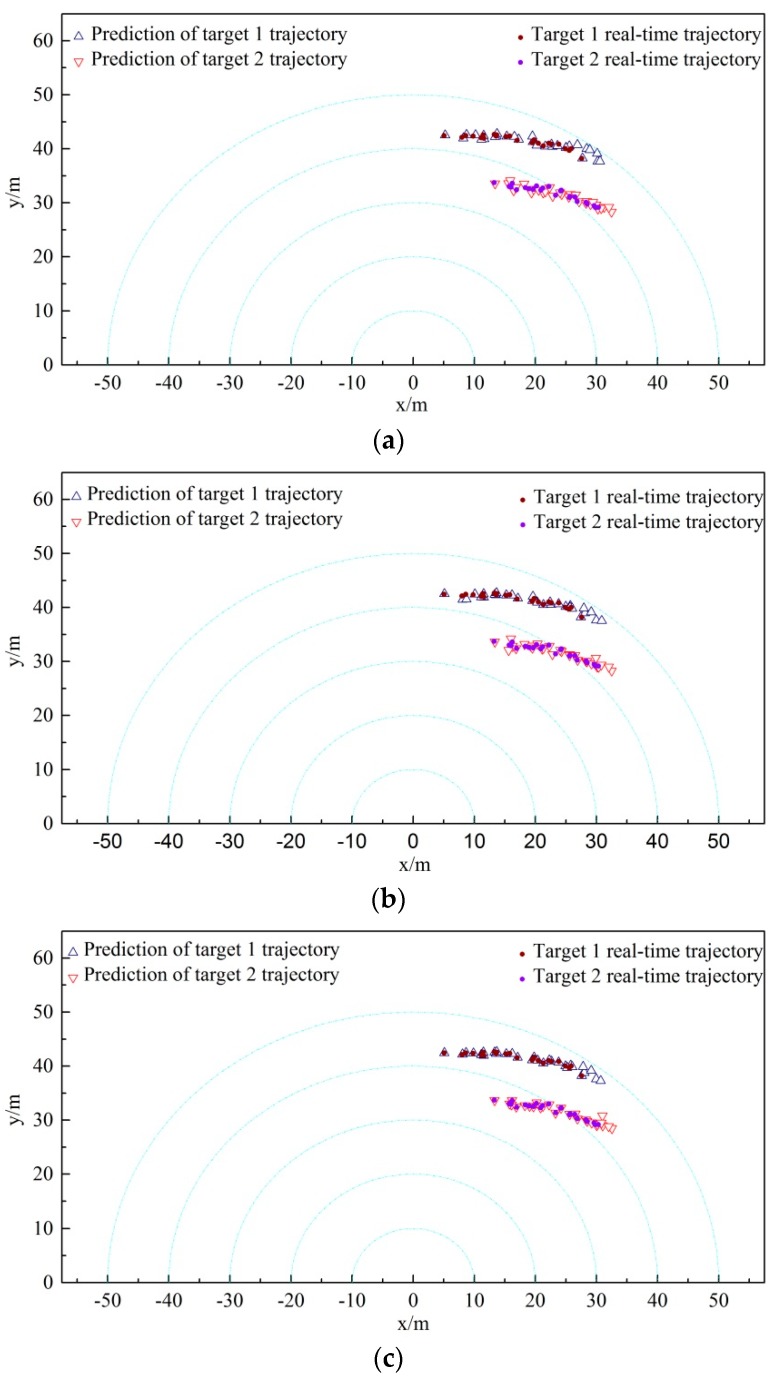
Non-cross-moving tracking diagram of multiple targets: (**a**) near neighbor data association (NNDA); (**b**) joint probabilistic data association (JPDA); (**c**) clustering cloud-like model (CCM).

**Figure 9:**
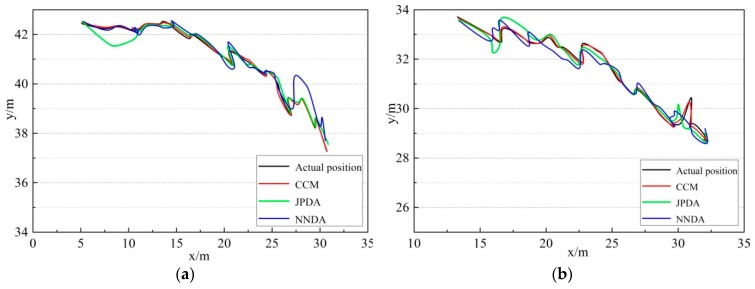
Actual-forecast comparison diagram of non-cross-moving targets: (**a**) target 1; (**b**) target 2.

**Figure 10:**
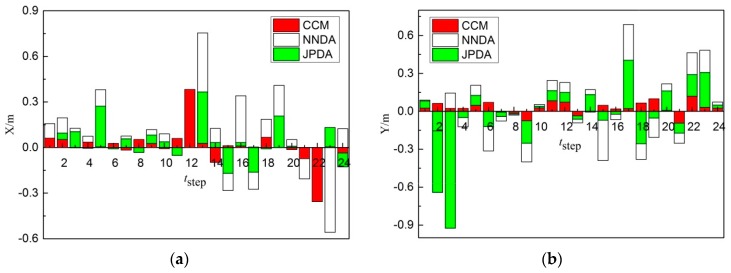
Errors comparison diagram of non-cross-moving target 1: (**a**) X-direction; (**b**) Y-direction.

**Figure 11:**
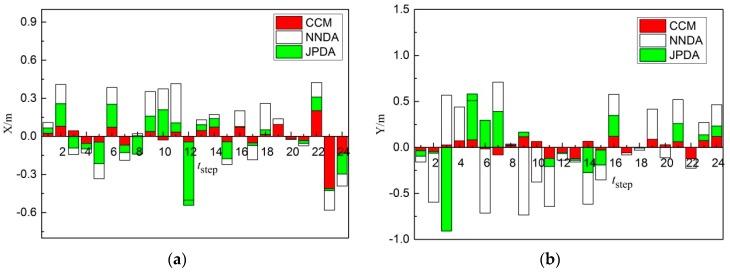
Errors comparison diagram of non-cross-moving target 2: (**a**) X-direction; (**b**) Y-direction.

The above-mentioned three kinds of data association algorithms are implemented to two non-cross-moving targets tracking correspondingly. The trajectory tracking results obtained from the three algorithms are listed in Figure 8. The comparison of results between the actual and the predicted positions of target 1 and 2 acquired from the three algorithms are shown in Figure 9, Figure 10 and Figure 11. Furthermore, the moving direction of target 1 and 2 is from left to right.

Through comparing the multiple targets trajectory tracking diagram and the actual-forecast comparison diagram obtained by three different correlation algorithms in Figure 8, we find that three algorithms can accomplish the tracking of the targets roughly when the two targets are non-cross-moving. The comparison results show that the CCM algorithm is more stable and the tracking error is less for the target 1 tracking. However, the JPDA algorithm deviates slightly from the actual trajectory of target 1 in the region at x∈[5,10], and the NNDA algorithm also diverges slightly in the region at x∈[27,30]. Therefore, the CCM algorithm can track the two targets accurately in the whole intervals with a better tracking performance.

As shown in Figure 9a, when comparing the targets trajectory tracking diagram obtained from the three different correlation algorithms, the actual and predicted trajectory comparison diagram shows that the above-mentioned algorithms can mainly complete the tracking of the two non-cross-moving targets. Nevertheless, for the CCM, the tracking of target 1 is more stable, and its error is less. The tracking of target 1 by JPDA in the region at x∈[5,10] is a little less than the target’s actual trajectory in the region at x∈[27,30], and the tracking of target 1 by NNDA deviates slightly from the actual trajectory. Meanwhile, as shown in Figure 9b, the tracking trajectory of target 2 for the CCM is closer to the actual trajectory than the other algorithms. The tracking of target 2 by JPDA in the region at x∈[15,19] and x∈[29,32] deviates from the actual trajectory, and the tracking of target 2 by NNDA deviates slightly from the actual trajectory in the first half of the region, and the whole tracking process is unstable. Compared with the above algorithms, the CCM algorithm can trace the targets more accurately during the whole tracking period.

As shown in Figure 10a, comparing the X-direction errors of non-cross-moving target 1 obtained from three different correlation algorithms, the tracking target 1 for the CCM is more stable and its error is less. Nevertheless, the error of the tracking of target 1 by NNDA in 24 steps is higher in general, and its tracking is unstable. The error of the tracking of target 1 by JPDA is slightly higher than CCM. As shown in Figure 10b, comparing the Y-direction errors of non-cross-moving target 1 obtained from the three different correlation algorithms, the tracking of target 1 for the CCM is more stable and its error is still less in 24 steps. The error of the tracking of target 1 by NNDA in 24 steps is higher than CCM, but better than JPDA. Therefore, the CCM algorithm can trace target 1 more accurately, and the error in X- and Y- direction is less than the other algorithms.

As shown in Figure 11a, comparing the X-direction errors of non-cross-moving target 2 obtained from the three different correlation algorithms, the tracking of target 2 for the CCM is more stable and its error is less. Nevertheless, the error of the tracking of target 2 by NNDA and JPDA in 24 steps is the same in general, and still higher than CCM. As shown in Figure 11b, comparing the Y-direction errors of non-cross-moving target 2 obtained from the three different correlation algorithms, the tracking of target 2 for the CCM is more stable, and its error is still less in 24 steps. The error of the tracking of target 2 by JPDA in 3–7 steps is higher. The error of the tracking of target 2 by NNDA in 24 steps is higher than JPDA, and its tracking is unstable in general. Therefore, the error of the CCM algorithm in the X and Y direction is less, and it can trace target 2 more accurately than the other algorithms.

### 4.2. Results and Analysis of Cross-Moving Experiment of Multiple Targets Experiment Using AUV

**Figure 12:**
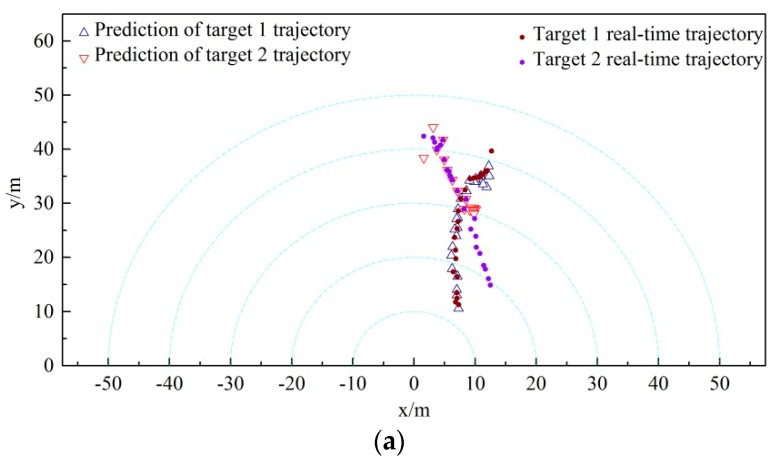

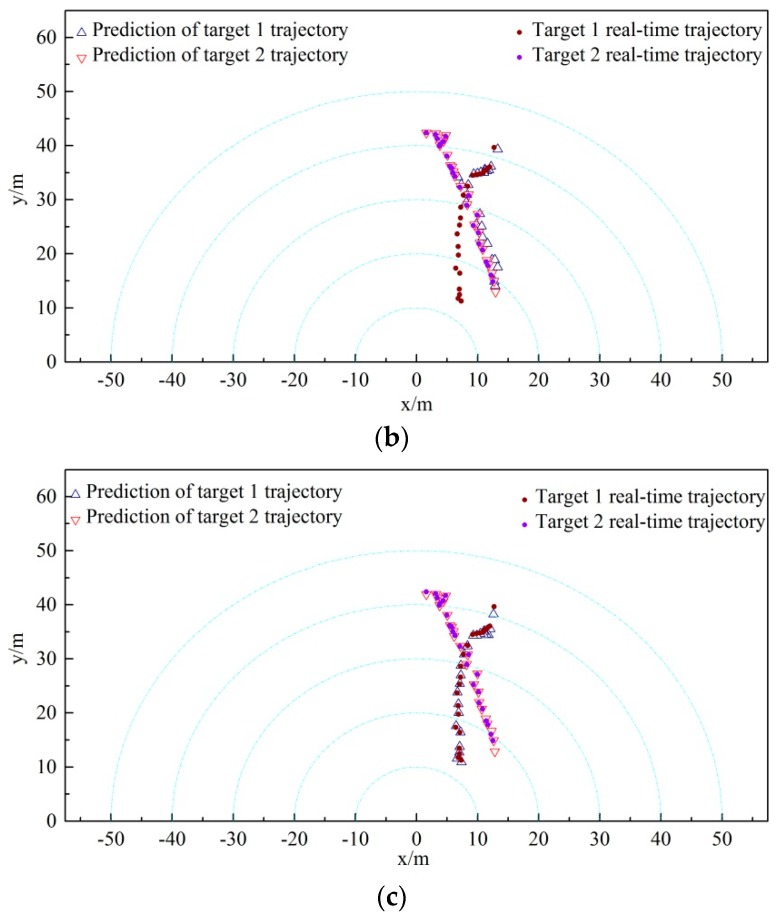
Cross-moving tracking diagram of multiple targets: (**a**) NNDA; (**b**) JPDA; (**c**) CCM.

**Figure 13:**
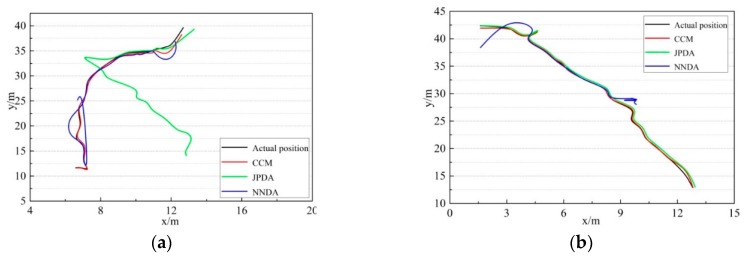
Actual-forecast comparison diagram of cross-moving targets: (**a**) target 1; (**b**) target 2.

**Figure 14:**
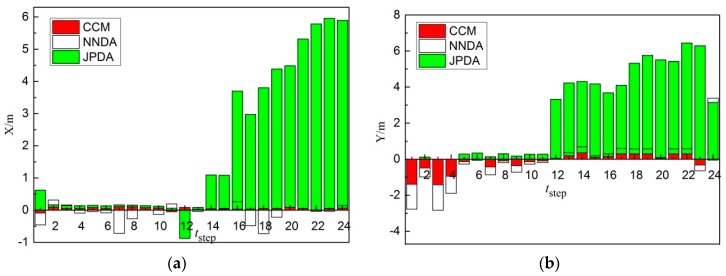
Errors comparison diagram of cross-moving target 1: (**a**) X-direction; (**b**) Y-direction.

**Figure 15:**
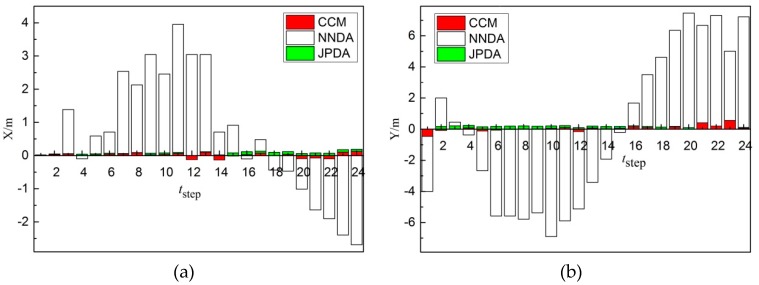
Errors comparison diagram of cross-moving target 2: (**a**) X-direction; (**b**) Y-direction.

The above-mentioned three kinds of data association algorithms were applied to two cross-moving targets tracking. The trajectory tracking results obtained from the three algorithms are shown in Figure 12. The comparison results between the actual and predicted trajectory of targets 1 and 2 acquired by the three algorithms are demonstrated in Figure 13, Figure 14 and Figure 15. The trajectory of target 1 is moving from the lower left direction to the upper right direction. Furthermore, the trajectory of target 2 is moving from the upper left direction to the lower right direction, slowly approaching the trajectory of target 1.

Comparing the multiple targets tracking diagram and the actual and predicted trajectory comparison diagram obtained from the three disparate correlation algorithms, we found that the JPDA and NNDA algorithm can nearly accomplish the tracking of target 1 when the two targets are cross-moving. The CCM algorithm, compared with the above algorithms, is more stable and the tracking error is less than that of target 1. Target 2 enters from the upper right, which approaches and intersects with target 1 in the region at y∈[25,30]. As illustrated in Figure 12a, the track loss of the NNDA algorithm occurs as a result of the error association with target 2 in the vicinity of the cross-over area. As shown in Figure 12b, the JPDA algorithm tracks the wrong target because of the error association in the vicinity of the cross-over area, and target 2 is mistaken as target 1 for tracking. 

As shown in Figure 13a, the actual and predicted trajectory comparison diagram of target 1 shows that the above-mentioned algorithms can mainly complete the tracking of the two non-cross-moving targets. For the CCM, the tracking trajectory of target 1 is more stable and closer to the actual trajectory among the three algorithms. Nevertheless, the tracking of target 1 by JPDA in the region of y∈[14,33] failed, and the tracking of target 1 by NNDA deviates slightly from the actual trajectory during the whole tracking period. Meanwhile, the actual and predicted trajectory comparison diagram of target 2 in Figure 13b shows that the above-mentioned algorithms can mainly complete the tracking of the two non-cross-moving targets. The tracking trajectory of target 2 of CCM and JPDA is close to the actual trajectory, and the whole tracking process is very stable. However, the tracking of target 2 by NNDA in the region at x∈[4,9] succeeded, but failed in another region. We can see that the CCM algorithm is capable of better performing the multi-target correlation and tracking the targets accurately during the whole period.

As shown in Figure 14a, comparing the X-direction errors of non-cross-moving target 1 obtained from three different correlation algorithms, the tracking of target 1 for the CCM is more stable and its error is less. The error of the tracking of target 1 by NNDA is slightly higher than CCM. Nevertheless, the error of the tracking of target 1 by JPDA in 13 steps is close to CCM, but the error in steps 14–24 is extremely high, so the tracking of JPDA in steps 14–24 failed. As shown in Figure 14b, when comparing the Y-direction errors of non-cross-moving target 1 obtained from the three different correlation algorithms, the tracking of target 1 for the CCM is more stable and its error is still less in 24 steps. The error of the tracking of target 1 by NNDA is slightly higher than CCM. The error of the tracking of target 1 by NNDA in 24 steps is slightly higher than CCM, but better than JPDA. The error of the tracking of target 1 by JPDA in steps 12–24 extremely high, so the tracking of JPDA in steps 12–24 failed. Therefore, the CCM algorithm can trace target 1 more accurately.

As shown in Figure 15a, comparing the X-direction errors of non-cross-moving target 2 obtained from the three different correlation algorithms, the tracking of target 2 for the CCM is more stable and its error is less. The error of the tracking of target 2 by JPDA in 24 steps is close to CCM in general. Nevertheless, the error of the tracking of target 2 by NNDA in 24 steps is extremely high, so the tracking of NNDA failed. As shown in Figure 15b, comparing the Y-direction errors of non-cross-moving target 2 obtained from the three different correlation algorithms, the tracking of target 2 for the CCM is more stable and its error is still less. The error of the tracking of target 2 by JPDA in 24 steps is close to CCM in general. Nevertheless, the error of the tracking of target 2 by NNDA in 24 steps is extremely high. Therefore, the error of the CCM algorithm in the X and Y direction is less and the CCM algorithm can trace target 2 more accurately in the three correlation algorithms.

## 5. Conclusions

To conclude, underwater multi-target tracking is an important issue of AUV trajectory planning during the period of detection and in barrier-avoiding. The mechanically scanned imaging sonar is fixedly mounted on AUVs for underwater targets tracking. Data association of underwater multiple targets is a very significant process in the advance of AUV tracking. However, conventional association algorithms of underwater multiple targets tracking data still have some shortcomings, including wrong tracking and tracking failure, especially during multiple targets that are in crosswise movement. Considering that the sample set for clustering here is the underwater target sonar image data, there is some random and fuzzy uncertainty. The cloud-like model is combined with the nearest neighbor algorithm to form a multi-target data association algorithm based on the clustering cloud-like model (CCM). AUV sea trial experiments were carried out to verify the effectiveness of the proposed algorithm. Experimental results indicate that both the NNDA and JPDA association occur as a result of the error association with other targets in the vicinity of the cross-over area, which is due to the error association in the neighborhood of the cross-over area. The proposed CCM algorithm leads to the right association results in both the non-crossover and crossover multi-target tracking experiments. Therefore, for future work, it is necessary to improve the real-time performance of the CCM algorithm for underwater targets tracking using the embedded system of AUVs.

## Figures and Tables

**Table 1:** Parameters of the Super SeaKing DST forward-looking sonar.

Property	Low Frequency	High Frequency
Frequency	CHIRP centered on 325 kHz	CHIRP centered on 650 kHz
Beam width	20° vertical, 3.0° horizontal	40° vertical, 1.5° horizontal
Pulse length	400 μs	200 μs
Maximum range	300 m	100 m
Minimum range	0.4 m	
Range resolution	approximately 15 mm (minimum)	
Source level	210 dB re 1 μPa at 1 m	
Mechanical resolutions	0.45°, 0.9°, 1.8°, and 3.6°	
Scanned sector	Variable up to 360°	
